# Merkel cell carcinoma with intramedullary spinal cord metastasis: a very rare clinical finding

**DOI:** 10.1002/ccr3.1516

**Published:** 2018-04-06

**Authors:** Tarek Haykal, Basim Towfiq

**Affiliations:** ^1^ Department of Internal Medicine Hurley Medical Center Michigan State University One Hurley Plaza Flint Michigan 48503

**Keywords:** Intramedullary, Merkel, metastasis, spinal cord

## Abstract

This clinical image teaches readers that a rare finding such as intramedullary spinal metastasis could exist even in the rarest tumors. It adds to the literature how it was managed. It also might reflect the improvement of our cancer treatment allowing us to follow patients longer to find those rare findings in rare tumors.

A 49‐year‐old female was diagnosed with stage 4 Merkel cell carcinoma of the vulva with pelvic and inguinal lymph node metastasis. She had received multiple chemotherapy and radiation therapy that failed, as only few months after diagnosis, the disease progressed and metastasized to the thoracic vertebrae and liver. She was waiting for a PET scan before starting her on immunotherapy as a final resort for treatment, but she presented with severe neck pain and left‐sided weakness of 10‐day duration. MRI of the spine revealed a 2.1 × 1.3 cm heterogeneous enhancing mass involving cervical spinal cord at C4–C5 level with expansion of the cord, that most likely be a metastasis of her disease, but another primary neoplastic disease could be ruled out (Fig. 1). The patient was treated with steroids, IV hydration, morphine PCA and also received 20 Gray of radiation in five treatments, and improved substantially initially. However, the patient despite immunotherapy kept deteriorating and is currently on hospice care.

**Figure 1:**
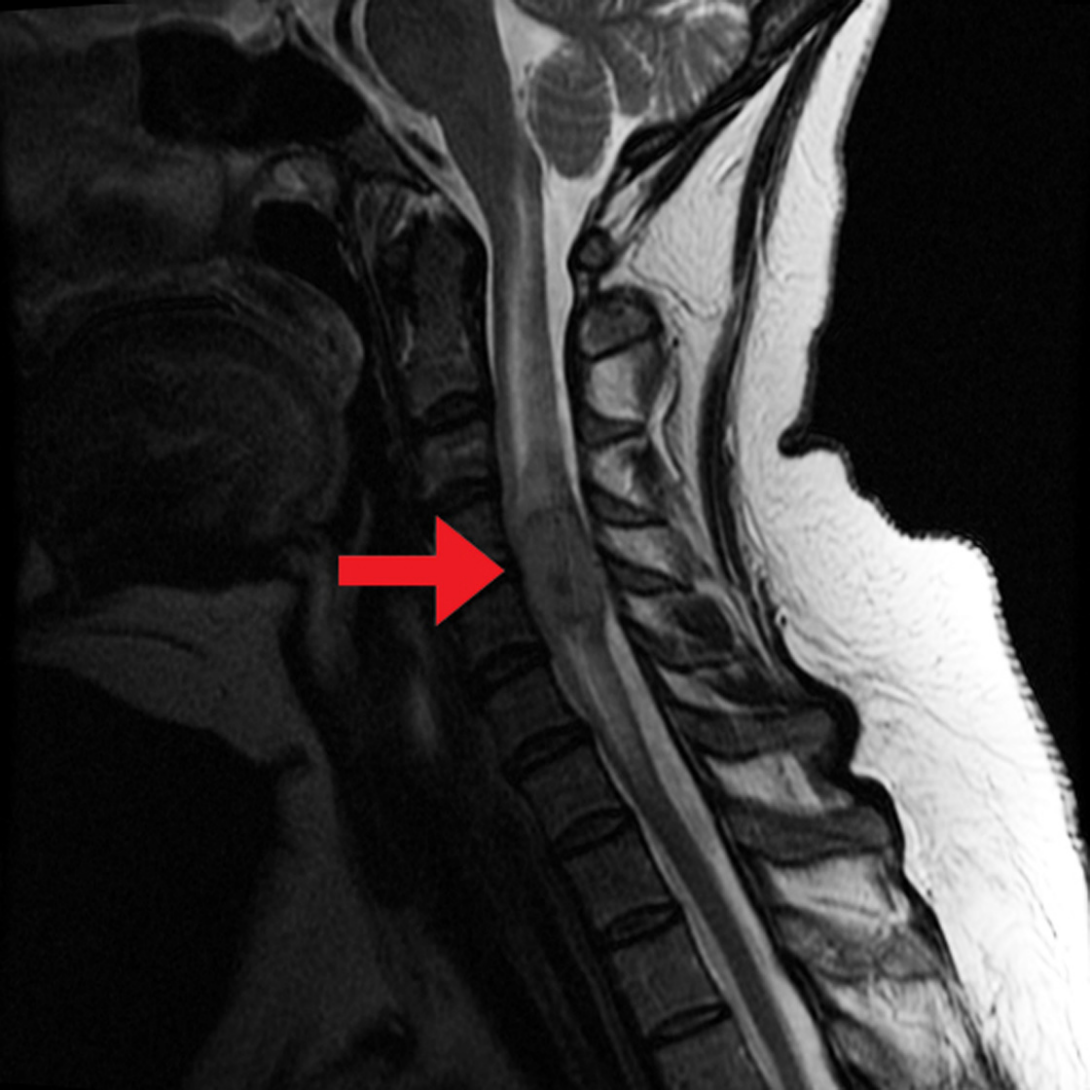
T2‐Weighted sequence of MRI of the cervical spine in sagittal view, showing a 2.1*1.3 centimeter heterogeneous enhancing solid intramedullary mass involving the cervical spine at C4‐C5 level with expansion of the cord.

Intramedullary spinal cord metastasis remains a very rare entity for malignancies that are not breast or lung in origin 1.

Merkel cell carcinoma is a rare cutaneous malignancy that is almost always locally invasive and causes metastasis frequently, especially to lymph nodes, liver, lungs, bone, and brain 2.

To the best of our knowledge, this is the second case in the literature for Merkel cell carcinoma to cause intramedullary spinal cord metastasis 2.

## Conflict of Interest

None declared.

## Authorship

TH: Primary Author, Original idea and manuscript writing. BT: Mentor and editor of the manuscript.
